# The effect of substitution on the unimolecular reaction rates of stabilized Criegee intermediates

**DOI:** 10.1039/d5cp04958j

**Published:** 2026-04-21

**Authors:** Aino Koskinen, Severi Juttula, Dominika Pasik, Nanna Myllys

**Affiliations:** a Department of Chemistry, University of Helsinki Helsinki 00014 Finland nanna.myllys@helsinki.fi; b Institute for Atmospheric and Earth System Research, University of Helsinki Helsinki 00014 Finland

## Abstract

Unimolecular reactions of stabilized Criegee intermediates (SCIs) with diverse substitution were studied using quantum chemical methods. The multiresonance electronic structure of SCIs was investigated by conducting both multireference and single reference benchmark calculations. A computationally inexpensive linear-scaling DLPNO-CCSD(T) method depicts SCIs sufficiently compared to other CCSD(T) levels of theory benchmarked in this study. Unimolecular reaction rate coefficients were calculated at the DLPNO-CCSD(T)/aug-cc-pVTZ//UM06-2X/aug-cc-pVTZ level of theory using lowest conformer transition state theory. We found several SCIs with slow unimolecular reactions and investigated how the molecular structure of a SCI affects its unimolecular reaction rate. Unimolecular structure–activity relationships were supplemented with reactivity trends for monocyclic saturated SCIs, conjugated cyclic SCIs, open chain aldehyde SCIs, and bicyclic monoterpene-derived SCIs. Structures with slow unimolecular reactions can react bimolecularly: long-lived SCIs are efficient oxidants of SO_2_ leading to sulfate aerosols and accretion reactions with oxygenated organics lead to low volatility organic compounds capable of secondary organic aerosol formation. This study highlights the different reactivity of SCIs, and their various pathways to impact Earth's radiation budget.

## Abstract 2

Unimolecular reactions of stabilized Criegee intermediates (SCIs) with diverse substitution were studied using quantum chemical methods.

## Graphical abstract

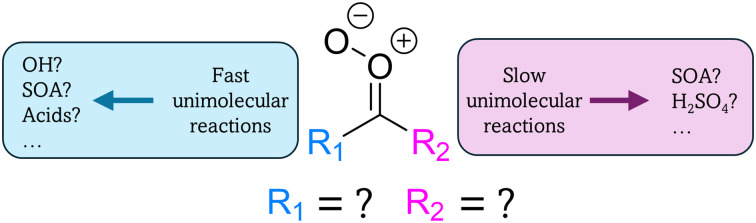

## 1. Introduction

One of the main loss channels of volatile organic compounds (VOCs) in the atmosphere is ozonolysis,^1^ a concerted [3+2] cycloaddition of ozone and alkenes. Ozonolysis produces primary ozonides (POZ), which further dissociate into a carbonyl compound and a carbonyl oxide, *i.e.* a Criegee intermediate (CI).^2^ As the formation of CIs is highly exothermic, CIs can rapidly decompose or become collisionally stabilized after their formation.^3–5^ Decomposition reactions of CIs produce multiple different fragments, such as OH radicals and acids.^5^

Collisionally stabilized Criegee intermediates (SCIs) can react both uni- and bimolecularly. In the atmosphere, approximately half of the SCIs formed from biogenic VOCs are decomposed *via* unimolecular decay and the rest are decomposed bimolecularly. Water monomer H_2_O and dimer (H_2_O)_2_ are responsible for the second largest quota of SCI loss.^5^ SCIs are important species in the troposphere for several reasons: they can oxidize SO_2_ to SO_3_ which is a key step in sulfuric acid and sulfate aerosol formation^6,7^ and affect HO_*x*_, NO_*x*_, and SO_*x*_ cycles.^8^ Sufficiently large SCIs that have a carbonyl functionality can also react intramolecularly to form secondary ozonides (SOZ), which have a role in secondary organic aerosol (SOA) formation.^9,10^

Unimolecular reaction rates are often very high, which limits how SCIs can react bimolecularly with other compounds.^5^ The atmospheric lifetime of SCIs is highly dependent on their substituents and possible isomers.^9^ Long-lived SCIs can oxidize SO_2_ leading to the formation of sulfate aerosols or they can react with oxygenated organics leading to low volatility organic compounds capable of forming SOA. The aim of this study is to investigate how the molecular structure of a SCI affects its lifetime and to find the most stable SCI structures, *i.e.* SCIs for which unimolecular reactions are slow.

There are multiple studies in which the unimolecular reaction rates of different SCIs have been investigated before. For example, Vereecken *et al.*^5,8^ have studied the effect of multiple functional groups and substituents on the unimolecular reaction rates of SCIs. Unimolecular reactions of open chain aldehyde SCIs with a carbon chain of five and six carbons have also been studied in the literature by Long *et al.*^10^ Unimolecular reactions of α-pinene-,^5^ β-pinene-,^11^ and sabinene-SCIs,^12^ atmospherically relevant monoterpene-derived SCIs, have been studied previously. Additionally, unimolecular reactions of SCIs with a benzene substituent^13^ and with a cyclohexane substituent^14^ have been studied before. This study provides systematic research on the effect of substitution of SCIs on the unimolecular reaction rates. The structure–activity relationships (SARs) drawn from the results help provide a broader perspective on the existing results and supplement the already existing SARs^5,8^ on the unimolecular reactions of SCIs.

A few long-lived SCI structures have already been reported in the literature. For example, Vereecken *et al.*^5^ reported *syn-t*Bu- and (*t*Bu)_2_-SCIs and Yu *et al.*^13^ reported a *syn*-ben-SCI to have slow unimolecular reactions. This is also the conclusion made in this study. In this study, more SCIs with slow unimolecular reactions are reported and their possible ability to contribute to aerosol formation is highlighted.

## 2. Computational details

### 2.1. Benchmark calculations

Because the molecular structure of a SCI is highly multi-resonance, we perform both multireference and single reference benchmark calculations to assess how well single reference methods are able to capture their multireference characteristic. Multireference calculations are performed at CASSCF, CASPT2, and DLPNO-NEVPT2 levels of theory and single reference calculations at several coupled cluster (CCSD(T)) levels of theory. Based on the benchmark calculations, we choose a suitable level of theory to lead accurate reaction rate coefficients.

**Fig. 1:**
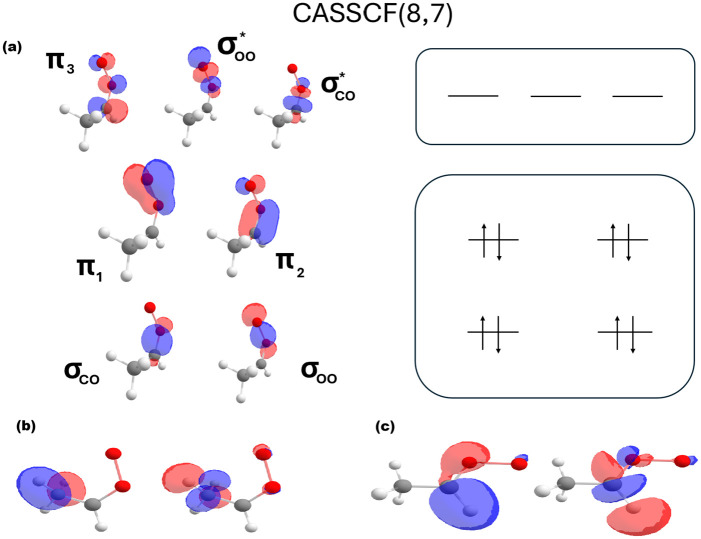
The active spaces used in multireference calculations. (a) The (8,7) active space, which corresponds to the σ- and π-system around the COO-moiety. (b) The additional orbitals included in the (1,4)-H-shift and (c) (1,3)-H-shift, resulting in the (10,9) space in each. Note that since the hydrogens are equivalent in the initial *syn*-conformer geometry, the resulting orbital includes both of them.

Multireference benchmark calculations were performed for *anti*-met-SCI and *syn*-met-SCI reactants and transition states for in-plane stereoisomerization, 1,4-H-shift (*syn*), 1,3-H-shift (*anti*), and 1,3-ring closure (*syn* and *anti*). The starting point of active spaces was one consisting of 8 electrons on 7 orbitals. This space corresponds to the σ- and π-system around the COO-moiety (Fig. 1), and it has been used previously to investigate the reactions of Criegee intermediates.^15,16^ However, it was noticed that for the in-plane-conformer change transition state to converge properly, we also had to include an 2s-type orbital from the two oxygen atoms. This can be justified by the fact that in this linear geometry the two σ-orbitals are conjugated. Additionally, we introduced the σ/*anti*-σ-orbital pair from the transferring hydrogen in the hydrogen transfer reactions. Therefore, the resulting active spaces are (8,7) for the ring closures, (10,8) for the conformer change, and (10,9) for the hydrogen shifts.

The CASSCF calculations were started from the UM06-2X/aug-cc-pVTZ geometry of the reactant, from which the MP2 natural orbitals were computed. The MP2 natural orbitals of the reactant were used as starting orbitals for the CASSCF calculation at the CASSCF/aug-cc-pVTZ level of theory. This was then followed by a frequency analysis at the same level of theory for the reactant. For the (1,4)-H-shift transition state, the optimized reactant orbitals obtained at the CASSCF/aug-cc-pVTZ level of theory were used as starting orbitals. For the other transition states, the MP2 natural orbitals were first computed, from which we then chose a set of orbitals corresponding to the same sets as in the reactant. The reason for this different treatment was that the orbitals obtained from using the starting geometry orbitals were too different from the expected ones, and thus they had to be recalculated. Transition states were optimized and their frequencies were calculated at the CASSCF/aug-cc-pVTZ level of theory.

CASPT2 calculations were performed at the CASPT2/aug-cc-pVTZ//CASSCF/aug-cc-pVTZ level of theory. Optimized reactant or transition state orbitals obtained at the CASSCF/aug-cc-pVTZ level of theory were used as starting orbitals for the reactants and transition states, respectively. Due to the high computational cost of CASPT2 calculations, geometries were not optimized at the CASPT2/aug-cc-pVTZ level of theory. Instead, the CASPT2 energies were obtained as single point energies at the CASPT2/aug-cc-pVTZ level of theory using the CASSCF-optimized geometries.

DLPNO-NEVPT2 calculations were performed similarly to the CASPT2 calculations as single point calculations at the DLPNO-NEVPT2/aug-cc-pVTZ//CASSCF/aug-cc-pVTZ level of theory. Again, optimized orbitals obtained at the CASSCF/aug-cc-pVTZ level of theory were used as starting orbitals. All multireference calculations were conducted using Orca 6.0.^17^

As multireference methods are computationally too expensive for most of the systems investigated in this study, several high-level single reference coupled cluster levels were also benchmarked for the same reactions as above. In single reference calculations, the SCI structures were treated as biradicals, *i.e.* open-shell singlet species. Single point energy (SPE) CCSD(T) calculations were performed at CCSD(T)-F12/cc-pVDZ,^18,19^ CCSD(T)-F12/cc-pVTZ,^18,19^ DLPNO-CCSD(T)-F12/cc-pVDZ,^19,20^ DLPNO-CCSD(T)-F12/cc-pVTZ,^19,20^ and DLPNO-CCSD(T)/aug-cc-pVTZ^19,21–23^ levels of theory.

### 2.2. Computational workflow

The workflow presented by Pasik *et al.*^24^ was used in this study to calculate reaction rate coefficients. A potential energy surface (PES) scan was performed to obtain the transition state (TS) for each reaction at the density functional theory (DFT) UM06-2X/6-31+G*^25,26^ level of theory. The scan was carried out starting from the reactant, which was optimized at the UM06-2X/6-31+G* level of theory prior to the PES scan.

**Fig. 2:**
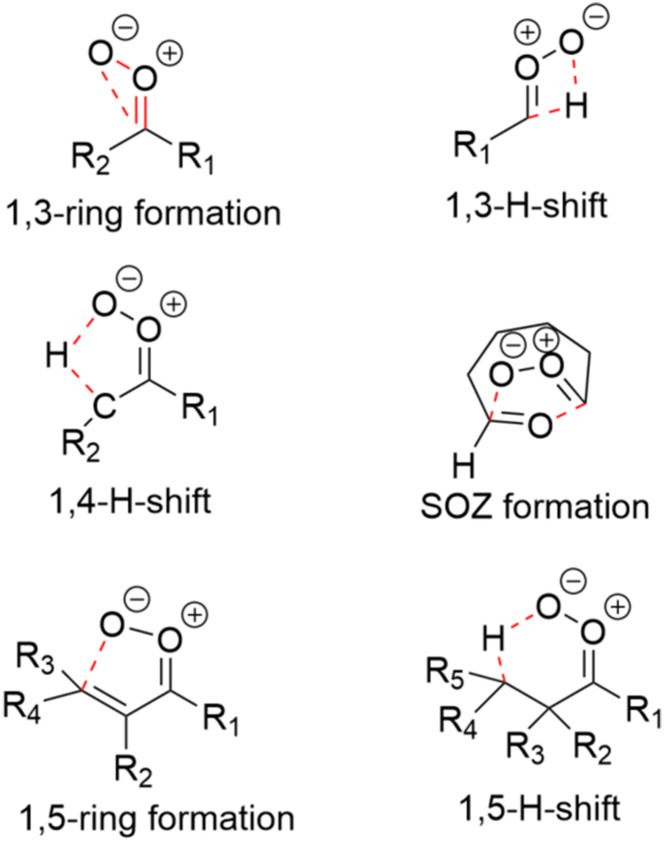
The bonds included in the TS formation were constrained during the conformational search with CREST and GOAT. Constrained bonds in each reaction are marked with red (dashed) lines.

After finding one TS and reactant conformer and optimizing them at the UM06-2X/6-31+G* level of theory, the conformer sampling for them was done using CREST^27^ and GOAT^17^ conformer sampling tools at the GFN2-xTB level of theory.^28^ To obtain the TS conformers with CREST or GOAT, the bonds included in the TS formation had to be constrained. The bonds to constrain in each of the reactions are depicted in Fig. 2. TS and reactant conformers found at the GFN2-xTB level of theory were optimized and their frequencies calculated at the UM06-2X/6-31+G* level of theory. TS structures were identified by one imaginary frequency corresponding to the correct vibration.

The lowest energy conformer of the reactant and the TS obtained at the UM06-2X/6-31+G* level of theory was optimized followed by a frequency calculation at the UM06-2X/aug-cc-pVTZ^19,23,25^ level of theory. The SPE corrections for the lowest energy reactant and the TS were carried out at the DLPNO-CCSD(T)/aug-cc-pVTZ level of theory based on our benchmark calculations. All the DFT calculations were carried out using Gaussian 16 software,^29^ and the CCSD(T) calculations were calculated with ORCA version 6.0.1.^17^

### 2.3. Unimolecular rate coefficients

As most of the structures in this study are fairly rigid and thus have one to few conformers, the lowest-conformer transition state theory (LC-TST)^30^ was utilized to calculate the reactant rate coefficients. The only SCI structures to have a significant number of conformers are the open chain structures with carbonyl functionalities. Due to the use of LC-TST, the rate coefficients of those structures are approximate. The reaction rate coefficients were calculated using eqn (1):^31^1
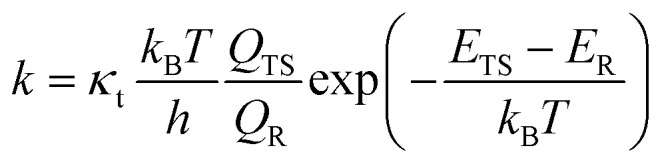
where *κ*_t_ is the quantum-mechanical tunneling coefficient, *k*_B_ is Boltzmann's constant, and *h* is Planck's constant. *Q*_TS_ is the partition function of the lowest-energy TS conformer and *Q*_R_ is the partition function for the lowest-energy reactant calculated at the UM06-2X/aug-cc-pVTZ level of theory. *E*_TS_ and *E*_R_ are the electronic DLPNO-CCSD(T)/aug-cc-pVTZ zero-point corrected energies of the lowest energy conformers of the TS and the reactant. Thus *E*_TS_–*E*_R_ is the energy barrier of the reaction.

*κ*_t_ was calculated for the H-shift reactions to account for the tunneling of hydrogen atoms. The tunneling occurs due to the low mass of the hydrogen.^32^ The tunneling coefficient was calculated using the one-dimensional asymmetric Eckart tunneling method.^33,34^ Forward and reverse intrinsic reaction coordinate calculations were performed for the lowest-energy TS conformer at the UM06-2X/6-31+G* level of theory. IRC calculation connects the TS to the reactant and product. IRC reactant and product structures were optimized at the UM06-2X/aug-cc-pVTZ level of theory, and the SPE corrections were carried out at the DLPNO-CCSD(T)/aug-cc-pVTZ level of theory. SPEs were zero-point corrected with the ZPEs obtained with UM06-2X/aug-cc-pVTZ. Imaginary frequencies from the UM06-2X/aug-cc-pVTZ and the energies of the IRC product and reactant were used to calculate the tunneling coefficient.

A higher DFT level of theory, UM06-2X/aug-cc-pVTZ was utilized in optimizing the lowest-energy conformers of the reactants and TSs due to the multireference characteristic of SCIs. UM06-2X/6-31+G* would very likely have been too low a theory level to yield sufficiently accurate geometries and zero-point energies for highly multireference SCIs. However, due to the high computational cost of UM06-2X/aug-cc-pVTZ, only the lowest-energy conformer was optimized even for reactants that had multiple reactant conformers (aldehyde-substituted SCIs). The use of LC-TST was validated by comparing rate coefficients obtained at DLPNO-CCSD(T)/aug-cc-pVTZ//UM06-2X/aug-cc-pVTZ to available theoretical rate coefficients taking multiple conformers into account and experimental rate coefficients. LC-TST and the aforementioned rate coefficients agree quite well (Table S3), which validates the use of LC-TST.

## 3. Results

### 3.1. Benchmark calculations

The differences in barrier energies of benchmarked reactions between the most computationally expensive single reference level of theory benchmarked in this study (CCSD(T)-F12/cc-pVTZ) and other CCSD(T) levels of theory are listed in Table 1. It can be seen from the results that all the CCSD(T) levels of theory yield extremely similar results for *syn*-met- and *anti*-met-SCIs. However, the deviation between DFT (UM06-2X/aug-cc-pVTZ) and CCSD(T) levels of theory is larger, as the mean absolute error of the difference of DFT and CCSD(T)-F12/cc-pVTZ levels of theory is 2.0 kcal mol^−1^. Since all of the CCSD(T) levels of theory yield extremely similar results, the least computationally expensive method, DLPNO-CCSD(T)/aug-cc-pVTZ, was chosen to calculate the SPE corrections on top of the DFT results in this study.

**Table 1:** Energy difference between barriers at the CCSD(T)-F12/cc-pVTZ and other CCSD(T) levels of theory (kcal mol^−1^). DFT energy barriers are calculated with UM06-2X/aug-cc-pVTZ. In the table, aVTZ means aug-cc-pVTZ, pVDZ cc-pVDZ, and pVTZ cc-pVTZ. Additionally, MAE stands for the mean absolute error and the reactions are labeled as follows: 1 = *syn*, 1,4-H-shift; 2 = *syn*, 1,3-ring closure; 3 = *syn-anti* stereoisomerization; 4 = *anti*, 1,3-H-shift; 5 = *anti*, 1,3-ring closure; 6 = *anti-syn* stereoisomerization

	Δ*E*_1_	Δ*E*_2_	Δ*E*_3_	Δ*E*_4_	Δ*E*_5_	Δ*E*_6_	MAE
DFT	−0.9	1.6	−3.6	0.3	2.3	−3.4	2.0
DLPNO/aVTZ	0.2	0.0	−0.5	−0.3	0.0	−0.5	0.2
DLPNO-F12/pVDZ	0.2	0.1	−0.5	−0.3	0.1	−0.4	0.3
DLPNO-F12/pVTZ	0.0	0.3	−0.3	−0.1	0.4	−0.3	0.2
F12/pVDZ	0.1	−0.2	−0.2	−0.1	0.0	−0.1	0.1

Table 2 shows energy barriers for the benchmarked unimolecular reactions calculated using the CASSCF, CASPT2, and DLPNO-NEVPT2 methods. Barriers obtained with DFT and CCSD(T) methods are compared to barriers computed with multireference methods in the SI (Tables S4–S6).

**Table 2:** Energy barriers of different unimolecular reactions of *syn*-met- and *anti*-met-SCIs calculated at the CASSCF, CASPT2, and DLPNO-NEVPT2 levels of theory. Zero-point energies used in the CASSCF, CASPT2, and DLPNO-NEVPT2 results were obtained at the CASSCF/aug-cc-pVTZ level of theory. Different reactions are labeled as follows: 1 = *syn*, 1,4-H-shift; 2 = *syn*, 1,3-ring closure; 3 = *syn*, in-plane stereoisomerization; 4 = *anti*, 1,3-H-shift; 5 = *anti*, 1,3-ring closure; 6 = *anti*, in-plane stereoisomerization. Their energy barriers are labelled as *E*_*i*_ where *i* is the number indicating the reaction type from the aforementioned list

	Δ*E*_1_	Δ*E*_2_	Δ*E*_3_	Δ*E*_4_	Δ*E*_5_	Δ*E*_6_
CASSCF	16.9	25.2	42.9	22.9	18.7	41.4
CASPT2	17.1	25.8	44.1	28.1	14.7	38.5
DLPNO-NEVPT2	19.8	24.6	39.4	12.7	16.1	33.4

For *syn*-met-SCI, the unimolecular rate coefficient corresponds to the rate coefficient of the dominant unimolecular reaction of it, 1,4-H-shift. For *anti*-met-SCI, the dominant unimolecular reaction is 1,3-ring closure. Thus, it is possible to draw some conclusions on the accuracy of multireference methods on 1,4-H-shift and 1,3-ring closure by comparing multireference results to empirical literature results. Table 3 shows the approximate reaction rate coefficients for the 1,4-H-shift using energy barriers obtained with CASSCF, CASPT2, and DLPNO-NEVPT2 methods.

**Table 3:** LC-TST reaction rate coefficients of 1,4-H-shift (*syn*-met-SCI) and 1,3-ring closure (*anti*-met-SCI) calculated using energy barriers obtained at the CASSCF/aug-cc-pVTZ, CASPT2/aug-cc-pVTZ//CASSCF/aug-cc-pVTZ, and DLPNO-NEVPT2/aug-cc-pVTZ//CASSCF/aug-cc-pVTZ levels of theory compared to the experimental reaction rate coefficients and reaction rate coefficients obtained at the DLPNO-CCSD(T)/aug-cc-pVTZ//UM06-2X/aug-cc-pVTZ level of theory. For rate coefficients calculated with CASSCF, CASPT2, and DLPNO-NEVPT2 methods, the partition functions used in the LC-TST formula were obtained at the UM06-2X/aug-cc-pVTZ level of theory and ZPE corrections at the CASSCF/aug-cc-pVTZ level of theory. Tunneling coefficients used in the rate coefficients of 1,4-H-shift were calculated at the DLPNO-CCSD(T)/aug-cc-pVTZ//UM06-2X/aug-cc-pVTZ level of theory

Method/Reaction	1,4-H-shift (s^−1^)	1,3-ring closure (s^−1^)
Experimental	290 ± 280	>3
CASSCF	70	0.2
CASPT2	50	100
DLPNO-NEVPT2	0.6	10
DLPNO-CCSD(T)	200	30

As can be seen from Table 3, CASSCF and CASPT2 yield approximate rate coefficients that are within the error margins of the experimental result (288 ± 275 s^−1^) for 1,4-H-shift. The rate coefficient obtained with DLPNO-NEVPT2, however, does not fall into the error margin of the experimental result. Thus it is possible to conclude that CASSCF and CASPT2 are able to portray 1,4-H-shift of *syn*-met-SCI more accurately than DLPNO-NEVPT2.

Although rate coefficients for 1,4-H-shift obtained at the CASSCF and CASPT2 levels of theory are within the error margins, the rate coefficient obtained at the DLPNO-CCSD(T)/aug-cc-pVTZ//UM06-2X/aug-cc-pVTZ level of theory is closer to the experimental value than the values obtained using multireference methods. Since DLPNO-CCSD(T) results align quite well with both experimental and multireference results, it can be concluded that the DLPNO-CCSD(T)/aug-cc-pVTZ//UM06-2X/aug-cc-pVTZ level of theory portrays the rate of 1,4-H-shift of *syn*-met-SCI sufficiently well.

For 1,3-ring closure, the rate coefficients in Table 3 highlight the importance of PT2 corrections to CASSCF energy. The difference between CASSCF and the experimental rate coefficient is at least one order of magnitude since the experimental value is given only as >3. Additionally, CASPT2 and DLPNO-NEVPT2 methods yield results of similar magnitude compared to results obtained with DLPNO-CCSD(T). This, along the fact that all of these rate coefficients are in line with the experimental one, indicates that differences in accuracies between these methods are small. The non-multireference characteristic of these reactions is also supported by the leading determinants in the CASSCF-level calculations always having coefficients larger than 0.9. Therefore, it can be concluded based on the available data that DLPNO-CCSD(T) is also able to portray the kinetics of 1,3-ring closure sufficiently well.

Finally, for the multireference calculations it should be noted that there is some uncertainty about the stereoisomerization transition state. The linear structure in the transition state geometry proved to be challenging. If the optimization criteria were tightened, then this structure would optimize to the other, out-of-plane rotation isomerization structure, or break apart completely. The former was not, however, investigated in this study, so it was not included. The final transition state geometry was eventually obtained by performing the transition state optimization by constraining the C–O bond length during optimization with tighter convergence criteria.

### 3.2. Unimolecular reactions

**Fig. 3:**
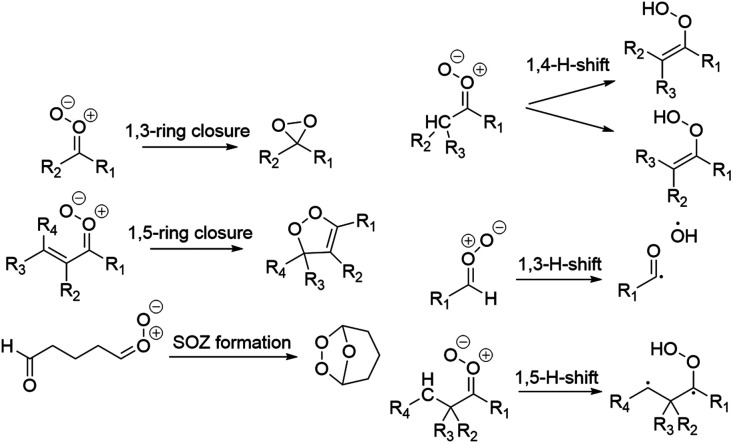
Reactions investigated in this study.

Unimolecular reactions studied for the SCIs depending on their structures were 1,3-ring closure, 1,5-ring closure, 1,3-H-shift, 1,4-H-shift, 1,5-H-shift, and SOZ formation (Fig. 3). The reactions to investigate were decided on based on structure–activity relationships (SAR)^5^ and the reactant structure (1,5-H-shift). Since the barrier of *syn-anti* stereoisomerization is high,^5^ the *syn* and *anti* isomers were treated as separate species.

The results and structures investigated for each structural class are presented in their respective subsections.

The 1,3-ring closure reaction rate coefficients were calculated for all of the studied SCI structures. The available H-shift (1,3-H-shift, 1,4-H-shift, or 1,5-H-shift) was calculated for almost every structure with a few exceptions: vinyl-1,5-H-shifts were not calculated for *syn*-pent-SCI or *syn*-ben-SCI because it is known that the reaction is very slow and the TS was hard to locate.

Vinyl-1,4-H-shifts were not calculated for cycpent-SCI or cychex-SCI because the zero-point corrected energy of the IRC product was higher in energy than the zero-point corrected energy of the TS. The electronic energy of the IRC products, however, were lower in energy than the electronic energy of the TS. The reaction is known to be slow due to the hydrogen shift from a conjugated cyclic structure, and thus it was not investigated further. For *syn* structures that were β unsaturated, reaction rate coefficients for 1,5-ring closure were also calculated. However, 1,5-ring closure was not investigated if the TS would have been extremely rigid and the reaction thus very slow (cycpent-SCI, cychex-SCI). The reaction rates of SOZ formation were calculated for structures with a carbonyl functionality. An exception is *anti*-ald3-SCI, for which the PES scan showed that SOZ formation does not happen since the structure merely forms a 1,3-ring. This is probably due to the short chain length, which does not allow the *anti* carbonyl oxide to form a cyclic SOZ. Additionally, the cyclic SOZ formed by *syn*-ald3-SCI decomposed into an open chain species, which was probably due to excessive ring strain in the relatively small cyclic SOZ.

#### 3.2.1. General reaction trends

There are clear trends in the reaction rates of 1,3-ring closure. If a non-bicyclic species has both *syn* and *anti* isomers, 1,3-ring closure is usually far faster for *anti* (approx. 13–400 s^−1^, Table S2) than for *syn* (approx. 10^−5^–10^−3^ s^−1^, Table S2) as has been stated in the literature before.^5^ The reason for this is the relative energy of the isomers: the highest energy isomer (*anti* or *syn*) reacts more rapidly and releases some of its energy in the transition state. This leads to lower energy barriers. The *syn* isomer is usually lower in energy than *anti*-SCIs with an α hydrogen.^5^ Additionally, the substitution of the β carbon in the *syn* position affects the reaction rate. The rate decreases when the steric hindrance in the substituent is increased.^5^ This can be seen in the 1,3-ring closure rate of *anti-t*Bu-SCI and *syn-t*Bu-SCI, which are 45.5 s^−1^ and 2.75 × 10^−3^ s^−1^, respectively (Table 4).

**Table 4:** Calculated energy barrier heights (Δ*E*^TS^, kcal/mol), Eckart tunneling coefficients (*κ*_t_) for H-shifts, and unimolecular LC-TST reaction rate coefficients (*k*_uni_, s^−1^) at 298 K for the fastest unimolecular reactions of the small saturated SCI structures

SCI	Reaction	Δ*E*^TS^	*κ*_t_	*k*_uni_
*anti*-met-SCI	1,3-ring closure	15.6	—	33.7
*syn*-met-SCI	1,4-H-shift	16.3	60	207
*anti-t*Bu-SCI	1,3-ring closure	15.2	—	45.4
*syn-t*Bu-SCI	1,3-ring closure	21.0	—	2.75 × 10^−3^
(*t*Bu)_2_-SCI	1,3-ring closure	20.0	—	2.08 × 10^−2^

The results show that 1,3-H-shift rate coefficients are almost the same for all of the *anti* SCIs (Table S2). As a conclusion, an alkyl substituent does not have a great effect on the rate of the 1,3-H-shift reaction as has been concluded before.^5^ Additionally, it can be concluded that open chain aldehyde substituents do not affect the rate of 1,3-H-shift either.

#### 3.2.2. Reaction trends for small saturated SCIs

**Fig. 4:**
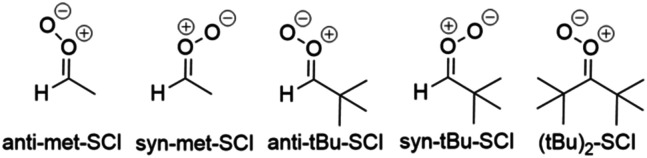
Small saturated SCIs investigated in this study.

Small saturated SCIs investigated in this study are depicted in Fig. 4 and the fastest unimolecular reactions for them are listed in Table 4.

The results are in line with the general trends: if the 1,4-H-shift is feasible for a *syn* stereoisomer, it is the fastest unimolecular reaction (*syn*-met-SCI). If there is no hydrogen in the β position, 1,3-ring closure is the fastest unimolecular reaction (*syn-t*Bu- and *syn*-(*t*Bu)_2_-SCIs). The extremely slow rate of 1,5-H-shift is not comparable to 1,3-ring closure (Table S2) as a high energy open shell biradical forms (Fig. 3). For *syn* isomers and twice substituted small SCIs, the rate of 1,3-ring closure is slow as shown in Table 4 and Table S2.

As discussed in general trends, 1,3-H-shift does not depend on alkyl substituents and is always slow. As the 1,3-ring closure is fast for *anti* stereoisomers, it is the fastest unimolecular reaction for the small saturated *anti* SCIs investigated in this study (*anti*-met- and *anti-t*Bu-SCIs).

#### 3.2.3. Reaction trends for monocyclic saturated SCIs

**Fig. 5:**
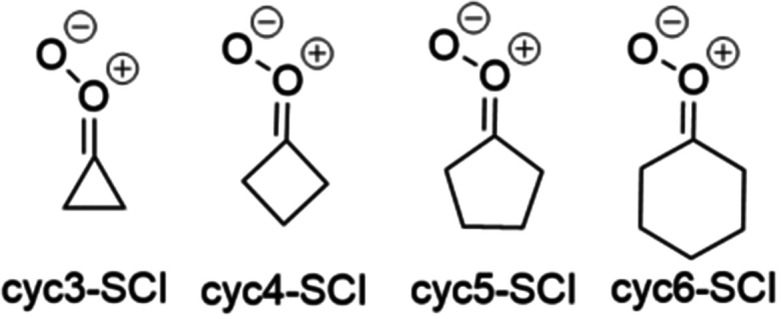
Monocyclic saturated SCIs investigated in this study.

Monocyclic saturated SCIs investigated in this study are shown in Fig. 5. The rate coefficients of their two possible unimolecular reactions, 1,4-H-shift and 1,3-ring closure, are presented in Table 5.

**Table 5:** Calculated energy barrier heights for 1,3-ring closure and 1,4-H-shift (Δ*E*^TS^, kcal mol^−1^) and the corresponding LC-TST rate coefficients at 298 K (*k*_uni_, s^−1^) for monocyclic SCIs. Eckart tunneling coefficients (*κ*_t_) are listed for H-shifts. Values in bold indicate the fastest unimolecular reaction for each of the monocyclic SCIs

SCI	Reaction	Δ*E*^TS^	*κ*_t_	*k*_uni_
cyc3-SCI	1,4-H-shift	25.5	90	4.60 × 10^−5^
	1,3-ring closure	15.9	—	9.91
cyc4-SCI	1,4-H-shift	18.6	50	1.04
	1,3-ring closure	16.7	—	0.986
cyc5-SCI	1,4-H-shift	16.7	50	79.6
	1,3-ring closure	19.2	—	3.88 × 10^−2^
cyc6-SCI	1,4-H-shift	14.5	40	1.40 × 10^3^
	1,3-ring closure	19.0	—	2.61 × 10^−2^

When comparing the rate coefficients for cyc3-SCI, cyc4-SCI, cyc5-SCI, and cyc6-SCI, it can be seen that the reaction rate seems to generally increase as the ring size increases. However, the four-membered ring (cyc4-SCI) is an exception: the reaction rate for that is slower than for the three-membered ring (cyc3-SCI). This can be explained, however, with the change in reaction mechanism: the 1,4-H-shift is slower than 1,3-ring closure for cyc3-SCI, but the 1,4-H-shift is faster than 1,3-ring closure for cyc4-SCI. The general trend is nevertheless clear: the rate of 1,3-ring closure decreases as the ring size increases, but the rate of 1,4-H-shift increases as the ring size increases (Table 5).

#### 3.2.4. Reaction trends for aldehyde substituted SCIs

**Fig. 6:**
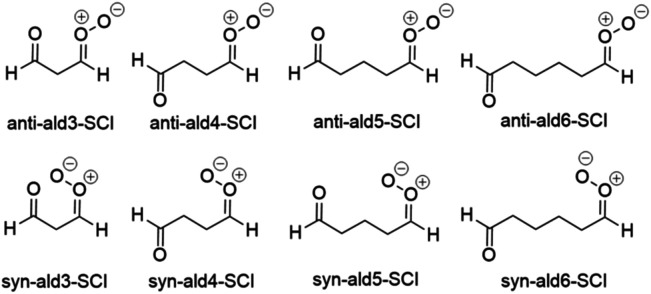
Aldehyde SCIs investigated in this study.

Fig. 6 shows the aldehyde SCIs investigated in this study. Open chain aldehyde SCIs have multiple conformers, and the number of conformers increases as the chain length increases and the structure becomes less rigid. In order to obtain the most accurate rate constant, all of the conformers would have to be taken into account by using the multi-conformer transition state theory (MC-TST).^30^ However, it has been shown before that the difference between the results obtained with MC-TST and LC-TST is approximately one order of magnitude or less.^30^ Indeed, our results with LC-TST align well with those of Long *et al.* where the multi-structural method with torsional anharmonicity (MS-T) was used.^10,35^ Our results are a maximum of four times larger than the results obtained by Long *et al.* (Table S3), which is in the error margin of used quantum chemical methods. Thus, LC-TST depicts even the structures with the largest number of conformers sufficiently well.

The fastest unimolecular reaction rates for aldehyde SCIs are listed in Table 6.

**Table 6:** Calculated energy barrier heights (Δ*E*^TS^, kcal mol^−1^), Eckart tunneling coefficients (*κ*_t_) for H-shifts, and unimolecular LC-TST reaction rate coefficients (*k*_uni_, s^−1^) at 298 K for the fastest unimolecular reactions of the aldehyde substituted SCI structures

SCI	Reaction	Δ*E*^TS^	*κ*_t_	*k*_uni_
*anti*-ald3-SCI	1,3-ring closure	15.6	—	18.0
*anti*-ald4-SCI	1,3-ring closure	14.8	—	61.9
*anti*-ald5-SCI	1,3-ring closure	14.0	—	230
*anti*-ald6-SCI	SOZ formation	5.1	—	8.26 × 10^7^
*syn*-ald3-SCI	1,4-H-shift	12.2	20	6.05 × 10^4^
*syn*-ald4-SCI	SOZ formation	3.7	—	9.01 × 10^8^
*syn*-ald5-SCI	SOZ formation	2.5	—	1.63 × 10^10^
*syn*-ald6-SCI	SOZ formation	3.0	—	5.78 × 10^8^

**Fig. 7:**
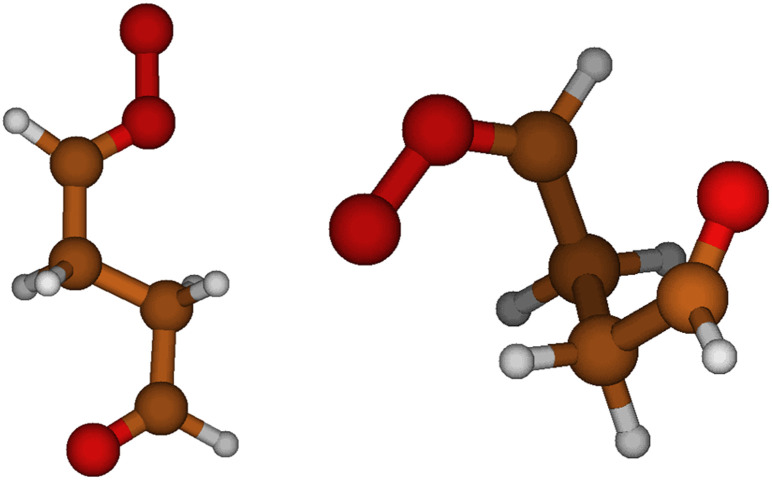
The differences between the SOZ formation rate coefficients of *anti* and *syn* structures can be explained by the folding of the lowest energy conformers of *anti* and *syn* structures. Because *syn* conformers can fold in a way that the carbonyl oxide is closer to carbonyl even with a relatively small number of carbon atoms in the carbon chain, *syn* structures react faster *via* SOZ formation than *anti* structures. This causes 25 orders of magnitude difference between SOZ formation rate coefficients of *anti*-ald4-SCI (left, *k*_SOZ_ = 3 × 10^−14^s^−1^) and *syn*-ald4-SCI (right, *k*_SOZ_ = 9 × 10^8^s^−1^).

The reaction coefficients of SOZ formation for the *syn*-SCIs are significantly greater than for *anti*-SCIs, which agrees with previous research.^10^ This is probably due to the ability of *syn* conformers to fold in a way that allows the carbonyl oxide to be closer to the carbonyl functional group even when the chain length is shorter (Fig. 7). The *anti* structures require a much longer chain to fold in the same way as the *syn* structures. Thus, the bonds in the *syn* aldehyde SCIs do not need to rotate as much as in *anti* aldehyde SCIs to form the TS. As this requires less energy, the barrier for *syn* SOZ formation is lower compared to *anti* SOZ formation. The reaction rate coefficients increase for *anti* aldehyde SCIs as the chain length increases, since the chain can fold in the same way as the *syn* aldehyde SCIs when the chain length increases. The results indicate that the SOZ formation becomes the dominant unimolecular reaction for open-chained aldehyde-SCIs as the number of carbon atoms connecting the functional groups is 4 for the *syn* isomer and 6 for the *anti* isomer.

Additionally, the rate of unimolecular SOZ formation for the carbonyl functionality containing SCIs generally increases as the chain length increases. This is caused by the decreasing rigidity of the TS structure. However, for the *syn*-ald6-SCI, the rate is slower than for *syn*-ald5-SCI and *syn*-ald4-SCI. This could be a discrepancy in our LC-TST results but as our results agree with previous research by Long *et al.* who used MS-T,^10^ it is an unlikely explanation. A possible reason stems from the way the lowest energy conformers of the *syn*-ald6-SCI reactant fold. Against the general trend, the lowest energy conformer of *syn*-ald6-SCI folds similarly to *anti*-ald4-SCI in Fig. 7, which decreases the reaction rate as the functional groups are far apart. Additionally, one of the lowest energy conformers of the *syn*-ald6-SCI reactant is folded in the same way as *syn*-ald4-SCI in Fig. 7. The energy difference of that conformer and the lowest energy conformer is approximately 0.3 kcal mol^−1^ at the UM06-2X/6-31+G* level of theory, which means that both conformers are present at the same time at 298 K and affect the reaction rate constant. This implies that in the conformer that is folded in the same way as *syn*-ald4-SCI in Fig. 7, the electromagnetic interactions between carbonyl oxide and carbonyl functional groups stabilize the reactant and thus contribute to decreasing the reaction rate.

#### 3.2.5. Reaction trends for conjugated cyclic SCIs

**Fig. 8:**
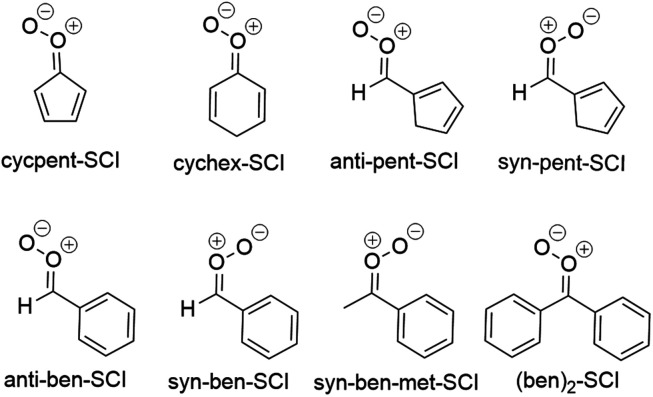
Conjugated cyclic SCIs investigated in this study.

The structures of conjugated cyclic SCIs investigated in this study are shown in Fig. 8 and the fastest unimolecular reactions for them are shown in Table 7.

**Table 7:** Calculated energy barrier heights (Δ*E*^TS^, kcal mol^−1^), Eckart tunneling coefficients (*κ*_t_) for H-shifts, and unimolecular LC-TST reaction rate coefficients (*k*_uni_, s^−1^) at 298 K for the fastest unimolecular reactions of the conjugated cyclic SCI structures

SCI	Reaction	Δ*E*^TS^	*κ*_t_	*k*_uni_
cycpent-SCI	1,3-ring closure	18.3	—	0.190
cychex-SCI	1,3-ring closure	17.2	—	1.07
*anti*-pent-SCI	1,3-ring closure	15.9	—	12.7
*syn*-pent-SCI	1,5-ring closure	14.4	—	63.7
*anti*-ben-SCI	1,3-ring closure	14.4	—	122
*syn*-ben-SCI	1,3-ring closure	22.5	—	1.85 × 10^−4^
*syn*-ben-met-SCI	1,3-ring closure	18.5	—	0.113
(ben)_2_-SCI	1,3-ring closure	16.3	—	4.22

The reaction rate for 1,3-ring closure is faster for cychex-SCI than cycpent-SCI. This is because the cycpent-SCI is better stabilized by resonance (4 resonance structures) than cychex-SCI (3 resonance structures). The role of resonance stabilization for 1,3-ring closures can also be seen in the results of *syn*-pent-SCI and *syn*-ben-SCI. As the *syn*-ben-SCI is better stabilized than *syn*-pent-SCI (4 resonance structures *vs.* 3 resonance structures), the reaction rate is slower for *syn*-ben-SCI than for *syn*-pent-SCI.

However, the same effect cannot be seen for 1,3-ring closure of met-ben-SCI, (ben)_2_-SCI or *anti*-pent- and *anti*-ben-SCIs. The results show that increasing substitution increases the 1,3-ring closure rate for *syn* benzene substituted SCIs. This can be seen from the 1,3-ring closure rates of *syn*-ben, *syn*-ben-met- and (ben)_2_-SCIs (1.85 × 10^−4^, 0.113, and 4.22 s^−1^, respectively). The effect of substitution is the same as for *syn-t*Bu-SCI and (*t*Bu)_2_-SCI, the rates of which are 2.75 × 10^−3^ and 2.08 × 10^−2^ s^−1^. Furthermore, as resonance stabilization increases for *anti* SCIs (*anti*-pent-SCI and *anti*-ben-SCI), the reaction rate of 1,3-ring closure increases by one order of magnitude. Resonance stabilization cannot explain these results.

Although it is usually a fast reaction for β unsaturated compounds, 1,5-ring closure is much slower for *syn*-pent-SCI, *syn*-ben-SCI, *syn*-met-ben-SCI, and (ben)_2_-SCI than for open-chain β unsaturated compounds studied before.^5,36,37^ Resonance stabilization explains some of the results. The resonance stabilization of *syn*-pent-SCI is the least significant of these SCIs, and the reaction rate coefficient for it is the largest (63 s^−1^). However, *syn*-ben-SCI, *syn*-met-ben-SCI, and (ben)_2_-SCI do not follow this trend. Instead, the effect of increasing substitution is more significant in this case as well. The rate coefficients of 1,5-ring closure increase as the substitution increases.

To conclude, more significant resonance stabilization decreases the rate of 1,3-ring and 1,5-ring closure if the size of the conjugated ring is altered. If substitution can be increased without altering the size of the conjugated ring, substitution plays a more significant role in defining the reaction rate than resonance stabilization. As substitution is increased, the reaction rate increases. However, these trends cannot be seen for *anti*-pent- and *anti*-ben-SCIs.

#### 3.2.6. Reaction trends for bicyclic monoterpene-derived SCIs

**Fig. 9:**
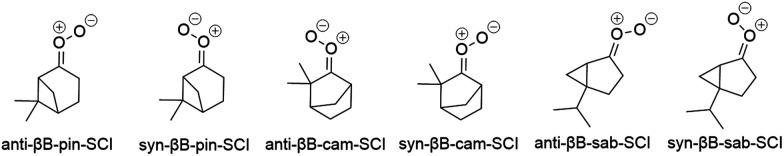
Bicyclic monoterpene derived SCIs investigated in this study.

SCIs derived from bicyclic monoterpenes that were investigated in this study are depicted in Fig. 9. Reactions available for these structures are 1,3-ring closure and 1,4-H-shift or 1,5-H-shift depending on the structure. Ring opening of the three-membered ring in sab-SCIs was also investigated, but no TS could be located. The reaction rate coefficients of 1,3-ring closure and the available H-shift for each of the structures are listed in Table 8. The structural feature that governs the rates of unimolecular reactions of bicyclic structures is the position of a bridgehead carbon. Bridgehead carbons are carbons connecting two rings in a bicyclic structure. Thus, bicyclic SCIs are named in this study based on the position of the bridgehead carbon in the structure. The prefix *syn*-β B means that there is a bridgehead carbon in the β position with respect to the outer oxygen in the carbonyl oxide in a structure. Similarly, the prefix *anti*-β B indicates that there is no β bridgehead carbon in the β position.

**Table 8:** Calculated energy barrier heights (Δ*E*^TS^, kcal mol^−1^), Eckart tunneling coefficients (*κ*_t_) for H-shifts, and unimolecular LC-TST reaction rate coefficients (*k*_uni_, s^−1^) at 298 K for both the 1,3-ring closure and available H-shift of bicyclic monoterpene-derived SCIs. Values in bold indicate the fastest unimolecular reaction for each of the bicyclic monoterpene-derived SCIs

SCI	Reaction	Δ*E*^TS^	*κ*_t_	*k*_uni_
*anti*-β B-pin-SCI	1,4-H-shift	15.2	40	835
	1,3-ring closure	20.9	—	1.55 × 10^−3^
*syn*-β B-pin-SCI	1,4-H-shift	24.9	4	7.07 × 10^−6^
	1,3-ring closure	17.0	—	1.55
*anti*-β B-cam-SCI	1,5-H-shift	35.6	20	3.23 × 10^−13^
	1,3-ring closure	18.6	—	9.93 × 10^−2^
*syn*-β B-cam-SCI	1,4-H-shift	30.2	3	6.99 × 10^−10^
	1,3-ring closure	16.3	—	3.05
*anti*-β B-sab-SCI	1,4-H-shift	15.0	40	937
	1,3-ring closure	17.1	—	1.14
*syn*-β B-sab-SCI	1,4-H-shift	23.7	6	1.54 × 10^−4^
	1,3-ring closure	16.7	—	2.18

The results show that 1,4-H-shifts are feasible if the hydrogen migrates from a non-bridgehead carbon. In that case 1,4-H-shift is the dominant unimolecular reaction over 1,3-ring closure (*anti*-β B-sab-SCI and *anti*-β B-pin-SCI). However, 1,4-H-shift is not feasible from a bridgehead carbon as has been concluded before in the literature.^11^ The reason behind this is the geometry of the singly occupied molecular orbital (SOMO) in the forming radical. SOMO is not stabilized by hyperconjugative effects in the same way in a tertiary bridgehead radical as in other tertiary radicals due to its geometry. Because its structure is rigid, the orientation of the SOMO cannot even be improved by rotation, which results in slow reaction rates.^11,38^ This effect can be seen in the 1,4-H-shift of *syn*-β B-pin-SCI, *syn*-β B-cam-SCI, and *syn*-β B-sab-SCI. 1,5-H-shift is again very slow as for the small saturated SCIs (*anti*-β B-cam-SCI).

The *anti-syn* trend for 1,3-ring closure described in the general trends is not conveyed by bicyclic SCIs. In such structures, the rate determining structural feature is a bridgehead carbon. If there is a bridgehead carbon in the β position, the 1,3-ring closure rates are faster than the rates of a bicyclic SCI without a β bridgehead carbon.

The reason behind this is the energy difference between bicyclic structures with a β bridgehead carbon and without it. Bicyclic structures with a bridgehead carbon in the β position are higher in energy than the other stereoisomer without a bridgehead carbon in the β position. Because the 1,3-ring closure is faster for higher energy conformers,^5^ the reaction rates increase. Table 9 shows the energy differences between the structures with a β bridgehead carbon and structures without it.

**Table 9:** Energy difference between bicyclic monoterpene-derived SCIs with a β bridgehead carbon and structures without it. Δ*E*_bridgehead_ (kcal mol^−1^) is the energy difference between the stereoisomer with a bridgehead carbon in the β position and the one without it (*E*_*syn*-βB_−*E*_*anti*-βB_). *E*_*syn*-βB_ and *E*_*anti*-βB_ are the DLPNO-CCSD(T)/aug-cc-pVTZ energies with ZPE corrections from UM06-2X/aug-cc-pVTZ

SCI	Δ*E*_bridgehead_
sab-SCI	1.1
cam-SCI	0.3
pin-SCI	2.3

This effect is also supported by the reaction rate coefficients of *anti*-β B-cam-SCI and *anti*-β B-pin-SCI that do not have a bridgehead carbon in the β position (9.93 × 10^−2^ s^−1^ and 1.56 × 10^−3^ s^−1^ respectively). The reaction rate coefficients of *anti*-β B-cam-SCI and *anti*-β B-pin-SCI are similar to the rate coefficient of the open chain (*t*Bu)_2_-SCI (2.08 × 10^−2^), which mimics the bicyclic structure. This supports the effect of the bridgehead position. However, the reaction rate coefficient of *anti*-β B-sab-SCI (1.14 s^−1^) is not close to those of *anti*-β B-cam-SCI and *anti*-β B-pin-SCI. Nonetheless, the rate for *anti*-β B-sab-SCI is still slower than the rate coefficient of 1,3-ring closure for *syn*-β B-sab-SCI (2.18 s^−1^) that has a bridgehead carbon in the β position.

**Fig. 10:**
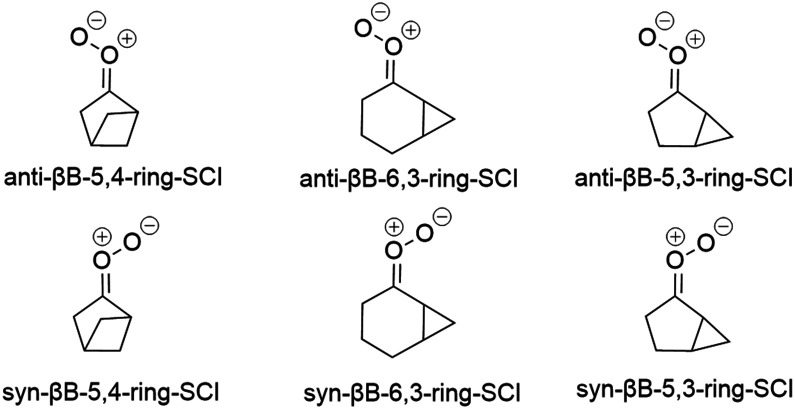
Structures with which the discrepancy in 1,3-ring closure reaction rates of sabinene-SCIs was investigated.

The reason behind the discrepancy in the results of sabinene-SCIs was further investigated. Sabinene-SCIs differ from camphene- and β-pinene-SCIs in ring size, the placement of the smaller ring, and substituents. Sabinene-SCIs have a five-membered ring whereas other bicyclic monoterpene-derived SCIs in this study have a six-membered ring. The second ring in sabinene does not connect the opposite sides in the larger ring as in pinene- and camphene-SCIs. Additionally, the isopropyl substituent differs from the substituents in pinene- and camphene-SCIs. To investigate the effect of each of the possible reasons, the reaction rate coefficient of 1,3-ring closure was calculated for *syn*-β B- and *anti*-β B-6,3-ring-SCIs, *syn*-β B- and *anti*-β B-5,4-ring-SCIs, and *syn*-β B- and *anti*-β B-5,3-ring-SCIs. The numbers in the names refer to the ring sizes in the structures and their structures are depicted in Fig. 10.

The results in Table 10 both validate the general trend for 1,3-ring closure of bicyclic structures further and explain the reason behind the discrepancy in the results of sabinene-SCIs. All of the investigated structures convey the same trend as bicyclic monoterpene derived SCIs: the structure without a β bridgehead carbon has a slower rate for 1,3-ring closure than the structure with a β bridgehead carbon. As 6,3-ring-SCIs do not convey the same trend as sabinene-SCIs, the ring placement (connecting opposite *vs.* neighboring carbons) cannot be the reason behind the discrepancy as 6,3-ring-SCIs would then align with the trend, too. The size of the bigger ring in the sabinene-SCIs cannot be the reason either: if it was, the rates of 5,4-ring-SCIs would convey the same trend as sab-SCIs. The results show that 5,3-ring-SCIs, *i.e.* sabinene-SCIs without the isopropyl substituent, align with the general trend that the structure without a β bridgehead carbon has a rate of at least one order of magnitude slower than the structure with one. That shows that the reason behind the discrepancy in the 1,3-ring closure rates of sabinene-SCIs is the isopropyl substituent.

**Table 10:** The rate constants of structures used to determine the cause of the discrepancy in the results of sabinene-SCIs

Structure	*k*_uni,*anti*-βB_	*k*_uni,*syn*-βB_
5,3-ring-SCI	0.713	2.43
6,3-ring-SCI	1.23 × 10^−2^	0.148
5,4-ring-SCI	8.64 × 10^−2^	24.1

### 3.3. Atmospheric implications

**Fig. 11:**
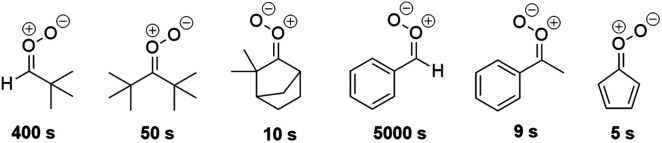
SCIs whose lifetimes are more than 1 second.

Several SCIs with slow unimolecular reactions were found. Structures with a lifetime longer than 1 second are portrayed in Fig. 11.

The unimolecular reaction rates limit how SCIs are able to react bimolecularly.^5^ Therefore structures with slow unimolecular reactions, *e.g.* SCIs depicted in Fig. 11, could react bimolecularly rather than unimolecularly.

SCIs can react bimolecularly with many different compounds. Such compounds include, for example, NO_*x*_, SO_2_, CO, water, water dimer, alcohols, aldehydes, and different acids.^39^ SCIs react the fastest with different acids, SO_2_, and NO_2_. Their rate constants are usually approximately 10^−10^, 10^−11^, and 10^−12^ cm^3^ molecule^−1^ s^−1^, respectively.^5,39^ Long-lived SCIs are very efficient oxidants of SO_2_ and have the potential to produce low-volatile accretion products with atmospheric vapors, leading to the formation of sulfate aerosols and secondary organic aerosols. This is a significant finding in atmospheric chemistry and may need to be accounted for when modelling the aerosol precursor formation under ozone-rich areas.

## 4. Conclusions

We investigated how the molecular structure of SCI affects its lifetime and found stable SCI structures that react bimolecularly rather than unimolecularly. *Syn*-Ben-SCI, *syn*-met-ben-SCI, *syn-t*Bu-SCI, (*t*Bu)_2_-SCI, *anti*-β B-cam-SCI, and cycpent-SCI had the slowest unimolecular reactions among the structures investigated. Their lifetimes in the atmosphere are longer than 1 second. Structures with slow unimolecular reactions could react bimolecularly with *e.g.* organic acids to form LVOCs that can contribute to SOA formation and with SO_2_ to form SO_3_ leading to sulfate aerosol formation. This highlights the different mechanisms of SCIs that can contribute to aerosol formation in the atmosphere. Both sulfate and organic aerosol particles are known to cool climate by scattering incoming radiation and by acting as cloud condensation nuclei.

The study also revealed some new additions to unimolecular structure–activity relationships regarding monocyclic saturated SCIs, open chain aldehyde SCIs, conjugated cyclic SCIs, and bicyclic monoterpene-derived SCIs. For monocyclic SCIs, increasing the size of the ring generally increases the reaction rate. For open chain aldehyde substituted SCIs the length of the carbon chain governs the unimolecular reaction rate. The rate is faster for *syn* aldehyde SCIs than *anti* aldehyde SCIs with the same number of carbons. Resonance stabilization and increasing substitution affect the unimolecular reaction rate of most conjugated cyclic SCIs. If the size of the conjugated ring is altered, resonance stabilization affects the reaction rate. If the size of the conjugated ring is not altered but the substitution is increased, the reaction rate also increases. For bicyclic monoterpene-derived SCIs, the position of a bridgehead carbon governs the reaction rate of 1,3-ring closure. This is a deviation from the general trend where the 1,3-ring closure is faster for *anti* stereoisomers compared to *syn* stereoisomers. The reaction rate of 1,3-ring closure for bicyclic SCIs is usually at least one order of magnitude larger for the *syn*-β B SCI compared to the *anti*-β B SCI.

We also conducted benchmark calculations to select the suitable level of theory for the SPE calculations of SCI reactions. The CCSD(T) calculations were performed at the CCSD(T)-F12/cc-pVDZ, CCSD(T)-F12/cc-pVTZ, DLPNO-CCSD(T)-F12/cc-pVDZ, DLPNO-CCSD(T)-F12/cc-pVTZ, and DLPNO-CCSD(T)/aug-cc-pVTZ levels of theory. All of the CCSD(T) methods yielded extremely similar results and thus it was possible to select a computationally inexpensive linear-scaling DLPNO-CCSD(T) method for the SPE corrections.

## Conflicts of interest

There are no conflicts to declare.

## Supplementary Material

CP-028-D5CP04958J-s001

## Data Availability

The optimized structures and calculation output files of all relevant compounds that support the findings of this manuscript will be available in the Zenodo repository (DOI: https://doi.org/10.5281/zenodo.17720296). Supplementary information (SI) is available. See DOI: https://doi.org/10.1039/d5cp04958j.
