# Supplementary material for: The effect of substitution on the unimolecular reaction rates of stabilized Criegee intermediates
Source: Phys Chem Chem Phys. 2026 Apr 21;28(17):10878–87. doi: 10.1039/d5cp04958j (PMC13096772; doi:10.1039/d5cp04958j)
Supplement: CP-028-D5CP04958J-s001 [file CP-028-D5CP04958J-s001.pdf]

Supplementary Information (SI) for Physical Chemistry Chemical Physics.
This journal is © the Owner Societies 2026

Supporting information for The Effect of Substitution on the Unimolecular Reaction Rates of Stabilized Criegee Intermediates

A. Koskinen, S. Juttula, D. Pasik, and N. Myllys

Fastest unimolecular reaction rates

The fastest unimolecular reaction rates at DLPNO-CCSD(T)/aug-cc-pVTZ//UM06-2X/aug-cc-pVTZ level of theory using LC-TST for all structures are listed in table S1. Structures that were used to investigate the discrepancy in sabinene-SCI results are not listed in any of the tables in the supplementary information.

Table S1: Calculated energy barrier heights (ΔE^{TS}; kcal/mol), Eckart tunneling coefficients (κ_t) for H-shifts, and unimolecular LC-TST reaction rate coefficients (k_{uni}, s^{-1}) at 298 K for the fastest unimolecular reactions of the SCI structures.

SCI	reaction	ΔE^{TS}	κ_t	k_{uni}
anti-met-SCI	1,3-ring closure	15.58	-	33.7
syn-met-SCI	1,4-H-shift	16.29	58.9	207
anti-tBu-SCI	1,3-ring closure	15.18	-	45.4
syn-tBu-SCI	1,3-ring closure	20.98	-	$2.75\cdot10^{-3}$
$(tBu)_2$-SCI	1,3-ring closure	20.03	-	$2.08\cdot10^{-2}$
cyc3-SCI	1,3-ring closure	15.86	-	9.91
cyc4-SCI	1,4-H-shift	18.64	46.5	1.04
cyc5-SCI	1,4-H-shift	16.73	48.1	79.6
cyc6-SCI	1,4-H-shift	14.52	35.6	$1.40\cdot10^{3}$
cycpent-SCI	1,3-ring closure	18.27	-	0.190
cychex-SCI	1,3-ring closure	17.19	-	1.07
anti-pent-SCI	1,3-ring closure	15.88	-	12.7
syn-pent-SCI	1,5-ring closure	14.38	-	63.7
anti-ben-SCI	1,3-ring closure	14.40	-	122
syn-ben-SCI	1,3-ring closure	22.50	-	$1.85\cdot10^{-4}$
syn-ben-met-SCI	1,3-ring closure	18.51	-	0.113
$(ben)_2$-SCI	1,3-ring closure	16.34	-	4.22
anti-ald3-SCI	1,3-ring closure	15.61	-	18.0
anti-ald4-SCI	1,3-ring closure	14.80	-	61.9
anti-ald5-SCI	1,3-ring closure	13.99	-	230
anti-ald6-SCI	SOZ formation	5.12	-	$8.26\cdot10^{7}$
syn-ald3-SCI	1,4-H-shift	12.16	21.7	$6.05\cdot10^{4}$
syn-ald4-SCI	SOZ formation	3.71	-	$9.01\cdot10^{8}$
syn-ald5-SCI	SOZ formation	2.46	-	$1.63\cdot10^{10}$
syn-ald6-SCI	SOZ formation	2.99	-	$5.78\cdot10^{8}$
anti-βB-cam-SCI	1,3-ring closure	18.65	-	$9.93\cdot10^{-2}$
syn-βB-cam-SCI	1,3-ring closure	16.31	-	3.05
anti-βB-pin-SCI	1,4-H-shift	15.24	42.4	835
syn-βB-pin-SCI	1,3-ring closure	16.98	-	1.55
anti-βB-sab-SCI	1,4-H-shift	15.03	36.7	937
syn-βB-sab-SCI	1,3-ring closure	16.70	-	2.18

All calculated unimolecular reaction rates

Reaction rate coefficients for all investigated reactions are listed in table S2. Reaction rate coefficients were calculated at DLPNO-CCSD(T)/aug-cc-pVTZ//UM06-2X/aug-cc-pVTZ level of theory utilizing LC-TST at 298 K.

Table S2: All calculated reaction coefficients for all investigated structures. For every reaction reaction barriers ΔE^{TS} (kcal/mol) and rate coefficients k_{uni} are listed. For H-shifts the tunneling coefficients κ_{t} are also listed. Table continues on the next page.

SCI	reaction	ΔE^{TS}	κ_{t}	k_{uni}
anti-met-SCI	1,3-H-shift	27.68	190	$9.67 \cdot 10^{-6}$
	1,3-ring closure	15.58	-	33.7
syn-met-SCI	1,4-H-shift	16.29	59	207
	1,3-ring closure	23.61	-	$3.51 \cdot 10^{-5}$
anti-tBu-SCI	1,3-H-shift	27.70	161	$7.12 \cdot 10^{-6}$
	1,3-ring closure	15.18	-	45.4
syn-tBu-SCI	1,5-H-shift	34.51	96	$1.09 \cdot 10^{-11}$
	1,3-ring closure	20.98	-	$2.75 \cdot 10^{-3}$
$(\mathrm{tBu})_2$-SCI	1,5-H-shift	34.34	23	$5.77 \cdot 10^{-12}$
	1,3-ring closure	20.03	-	$2.08 \cdot 10^{-2}$
cyc3-SCI	1,4-H-shift	25.55	86	$4.60 \cdot 10^{-5}$
	1,3-ring closure	15.86	-	9.91
cyc4-SCI	1,4-H-shift	18.64	47	1.04
	1,3-ring closure	16.65	-	0.986
cyc5-SCI	1,4-H-shift	16.73	48	79.6
	1,3-ring closure	19.16	-	$3.88 \cdot 10^{-2}$
cyc6-SCI	1,4-H-shift	14.52	36	$1.40 \cdot 10^{3}$
	1,3-ring closure	19.04	-	$2.61 \cdot 10^{-2}$
cycpent-SCI	1,3-ring closure	18.27	-	0.190
cychex-SCI	1,3-ring closure	17.19	-	1.07
anti-pent-SCI	1,3-H-shift	28.76	182	$1.03 \cdot 10^{-6}$
	1,3-ring closure	15.88	-	12.7
syn-pent-SCI	1,3-ring closure	21.17	-	$1.81 \cdot 10^{-3}$
	1,5-ring closure	14.38	-	63.7
anti-ben-SCI	1,3-H-shift	28.48	172	$1.51 \cdot 10^{-6}$
	1,3-ring closure	14.40	-	122
syn-ben-SCI	1,3-ring closure	22.50	-	$1.85 \cdot 10^{-4}$
	1,5-ring closure	23.26	-	$1.79 \cdot 10^{-5}$
syn-ben-met-SCI	1,3-ring closure	18.51	-	0.113
	1,5-ring closure	21.00	-	$1.33 \cdot 10^{-3}$
$(\mathrm{ben})_2$-SCI	1,3-ring closure	16.34	-	4.22
	1,5-ring closure	19.91	-	$6.02 \cdot 10^{-3}$

Table S2: (continued) All calculated reaction coefficients for all structures for which all of the possible or feasible reactions were investigated. For every reaction reaction barriers ΔE^{TS} (kcal/mol) and rate coefficients k_{uni} are listed. For H-shifts the tunneling coefficients κ_t are also listed. [1]=product is a open chain species and not cyclic

SCI	reaction	ΔE^{TS}	κ_t	k_{uni}
anti-ald3-SCI	1,3-H-shift	28.39	242	$1.89 \cdot 10^{-6}$
	1,3-ring closure	15.61	-	18.0
anti-ald4-SCI	1,3-H-shift	27.79	176	$5.16 \cdot 10^{-6}$
	1,3-ring closure	14.80	-	61.9
	SOZ formation	33.83	-	$3.05 \cdot 10^{-14}$
anti-ald5-SCI	1,3-H-shift	27.84	156	$3.69 \cdot 10^{-6}$
	1,3-ring closure	13.99	-	230
	SOZ formation	14.98	-	1.49
anti-ald6-SCI	1,3-H-shift	28.68	140	$2.63 \cdot 10^{-6}$
	1,3-ring closure	13.93	-	396
	SOZ formation	5.12	-	$8.26 \cdot 10^{7}$
syn-ald3-SCI	1,4-H-shift	12.16	22	$6.05 \cdot 10^{4}$
	1,3-ring closure	23.26	-	$5.09 \cdot 10^{-5}$
	SOZ formation[1]	13.35	-	113
syn-ald4-SCI	1,4-H-shift	17.49	50	31.9
	1,3-ring closure	23.11	-	$4.09 \cdot 10^{-5}$
	SOZ formation	3.71	-	$9.01 \cdot 10^{8}$
syn-ald5-SCI	1,4-H-shift	16.46	43	734
	1,3-ring closure	21.56	-	$1.01 \cdot 10^{-3}$
	SOZ formation	2.46	-	$1.63 \cdot 10^{10}$
syn-ald6-SCI	1,4-H-shift	15.81	43	309
	1,3-ring closure	23.34	-	$1.18 \cdot 10^{-5}$
	SOZ formation	2.99	-	$5.78 \cdot 10^{8}$
anti-βB-cam-SCI	1,5-H-shift	35.64	16	$3.23 \cdot 10^{-13}$
	1,3-ring closure	18.65	-	$9.93 \cdot 10^{-2}$
syn-βB-cam-SCI	1,4-H-shift	30.24	3	$6.99 \cdot 10^{-10}$
	1,3-ring closure	16.31	-	3.05
anti-βB-pin-SCI	1,4-H-shift	15.24	42	835
	1,3-ring closure	20.93	-	$1.55 \cdot 10^{-3}$
syn-βB-pin-SCI	1,4-H-shift	24.88	4	$7.07 \cdot 10^{-6}$
	1,3-ring closure	16.98	-	1.55
anti-βB-sab-SCI	1,4-H-shift	15.03	37	937
	1,3-ring closure	17.07	-	1.14
syn-βB-sab-SCI	1,4-H-shift	23.68	6	$1.54 \cdot 10^{-4}$
	1,3-ring closure	16.70	-	2.18

Unimolecular reaction coefficients in this study compared to literature values

Unimolecular reaction coefficients at 298 K acquired at DLPNO-CCSD(T)/aug-cc-pVTZ//UM06-2X/aug-cc-pVTZ level of theory utilizing LC-TST compared to selected theoretical and/or experimental values in the literature are listed in table S3.

Table S3: The results obtained in this study compared to results in the literature. All of the results in the literature for anti-met-SCI and syn-met-SCI are not listed in this table. Theoretical results from the literature are marked with T and experimental with E.

SCI	k_{uni}	k_{uni}, literature	reference
anti-met-SCI	33.7	T: 55.4	Long et al. 2016[1]
		T: 104	Vereecken et al. 2017[2]
		E: $>$ 3*	Berndt et al. 2015[3]
syn-met-SCI	207	E: 288±275	Newland et al. 2015[4]
		E+T**: 150^{+176}_{-81}	Robinson et al. 2022[5]
		T: 166	Fang et al. 2016[6]
		T: 328	Long et al. 2016[1]
		T: 257	Vereecken et al. 2017[2]
anti-tBu-SCI	45.4	T: 111	Vereecken et al. 2017[2]
syn-tBu-SCI	$2.75 \cdot 10^{-3}$	T: $1.0 \cdot 10^{-2}$	Vereecken et al. 2017[2]
$(tBu)_2$-SCI	$2.08 \cdot 10^{-2}$	T: $7.5 \cdot 10^{-2}$	Vereecken et al. 2017[2]
cyc3-SCI	9.91	-	-
cyc4-SCI	1.04	-	-
cyc5-SCI	79.6	-	-
cyc6-SCI	1400	E: 1998±147	Peltola et al. 2024[7]
cycpent-SCI	0.190	-	-
cychex-SCI	1.07	-	-
anti-pent-SCI	12.7	-	-
syn-pent-SCI	63.7	-	-
anti-ben-SCI	122	T: 141.85	Yu et al. 2025[8]
syn-ben-SCI	$1.85 \cdot 10^{-4}$	T: $4.22 \cdot 10^{-4}$	Yu et al. 2025[8]
syn-ben-met-SCI	0.113	-	-
$(ben)_2$	4.22	-	-
anti-ald3-SCI	18.0	-	-
anti-ald4-SCI	61.9	-	-
anti-ald5-SCI	230	T: 194	Long et al. 2019[9]
anti-ald6-SCI	$8.26 \cdot 10^{7}$	T: $2.44 \cdot 10^{7}$	Long et al. 2019[9]
syn-ald3-SCI	$6.05 \cdot 10^{4}$	-	-
syn-ald4-SCI	$9.01 \cdot 10^{8}$	-	-
syn-ald5-SCI	$1.63 \cdot 10^{10}$	T: $7.14 \cdot 10^{9}$	Long et al. 2019[9]
syn-ald6-SCI	$5.78 \cdot 10^{8}$	T: $3.24 \cdot 10^{8}$	Long et al. 2019[9]
anti-cam-SCI	3.05	-	-
syn-cam-SCI	$9.93 \cdot 10^{-2}$	-	-
anti-pin-SCI	835	T: 375	Vereecken et al. 2017[2]
syn-pin-SCI	1.55	T: 2.0	Vereecken et al. 2017[2]
anti-sab-SCI	937	T: 2740	Wang et al. 2017[10]
syn-sab-SCI	2.18	T: 0.97	Wang et al. 2017[10]

*at 297 K, **Fit to experimental results using Master Equation Solver for Multi-Energy well Reactions (MESMER)

Reaction barriers obtained with multireference calculations compared to DFT and CCSD(T) barriers

Unimolecular reaction barriers acquired at CASSCF, CASPT2, and DLPNO-NEVPT2 levels of theory compared to barriers obtained with DFT UM06-2X/aug-cc-pVTZ (Table S4) and CCSD(T)-F12/cc-pVTZ levels of theory (Table S5). As CCSD(T)-F12/cc-pVTZ is the most computationally expensive CCSD(T) level of theory benchmarked in this study, multireference methods were compared to the results obtained with it. Additionally, the differences in the barriers obtained with DLPNO-CCSD(T)/aug-cc-pVTZ and different multireference methods are presented in Table S6. This was done as the reaction rate coefficients in this study were calculated at DLPNO-CCSD(T)/aug-cc-pVTZ//UM06-2X/aug-cc-pVTZ level of theory.

Table S4: The difference between energy barriers obtained with DFT UM06-2X/aug-cc-pVTZ and used multireference methods (CASSCF, CASPT2, and DLPNO-NEVPT2). The reactions are numbered as follows: 1=syn, 1,4-H-shift; 2=syn, 1,3-ring closure; 3=syn, in-plane stereoisomerization; 4=anti, 1,3-H-shift; 5=anti, 1,3-ring closure; 6=anti, in-plane stereoisomerization. MAE stands for mean absolute error. Zero-point energies used in the CASSCF, CASPT2, and DLPNO-NEVPT2 results were obtained at CASSCF/aug-cc-pVTZ level of theory.

	ΔE_1	ΔE_2	ΔE_3	ΔE_4	ΔE_5	ΔE_6	MAE
CASSCF	1.6	0.0	5.1	-5.3	1.0	7.0	3.3
CASPT2	1.8	0.5	6.4	-0.1	-3.0	4.0	2.6
DLPNO-NEVPT2	4.6	-0.7	1.7	0.2	-5.0	1.1	2.2

Table S5: The difference between energy barriers obtained with CCSD(T)-F12/cc-pVTZ and used multireference methods (CASSCF, CASPT2, and DLPNO-NEVPT2). The reactions are numbered as follows: 1=syn, 1,4-H-shift; 2=syn, 1,3-ring closure; 3=syn, stereoisomerization; 4=anti, 1,3-H-shift; 5=anti, 1,3-ring closure; 6=anti, stereoisomerization. MAE stands for mean absolute error. Zero-point energies used in the CASSCF, CASPT2, and DLPNO-NEVPT2 results were obtained at CASSCF/aug-cc-pVTZ level of theory.

	ΔE_1	ΔE_2	ΔE_3	ΔE_4	ΔE_5	ΔE_6	MAE
CASSCF	0.8	1.7	1.5	-5.0	3.1	3.5	2.6
CASPT2	1.0	2.2	2.8	0.2	-0.9	0.6	1.3
DLPNO-NEVPT2	3.8	1.0	-1.9	0.5	2.9	-4.5	2.4

Table S6: The difference between energy barriers obtained with DLPNO-CCSD(T)/aug-cc-pVTZ and used multireference methods (CASSCF, CASPT2, and DLPNO-NEVPT2), The reactions are numbered as follows: 1=syn, 1,4-H-shift; 2=syn, 1,3-ring closure; 3=syn, stereoisomerization; 4=anti, 1,3-H-shift; 5=anti, 1,3-ring closure; 6=anti, stereoisomerization. MAE stands for mean absolute error. Zero-point energies used in the CASSCF, CASPT2, and DLPNO-NEVPT2 results were obtained at CASSCF/aug-cc-pVTZ level of theory.

	ΔE_1	ΔE_2	ΔE_3	ΔE_4	ΔE_5	ΔE_6	MAE
CASSCF	0.6	1.7	2.0	-4.8	3.0	4.0	2.7
CASPT2	0.8	2.1	3.2	0.5	-0.9	1.1	1.5
DLPNO-NEVPT2	3.6	1.0	-1.4	0.8	-2.9	-4.0	2.3

References

[1] B. Long, J. L. Bao and D. G. Truhlar, Atmospheric Chemistry of Criegee Intermediates: Unimolecular Reactions and Reactions with Water, *Journal of the American Chemical Society*, 2016, **138**, 14409–14422.

[2] L. Vereecken, A. Novelli and D. Taraborrelli, Unimolecular decay strongly limits the atmospheric impact of Criegee intermediates, *Physical Chemistry Chemical Physics*, 2017, **19**, 31599–31612.

[3] T. Berndt, R. Kaethner, J. Voigtländer, F. Stratmann, M. Pfeifle, P. Reichle, M. Sipilä, M. Kulmala and M. Olzmann, Kinetics of the unimolecular reaction of CH_2 OO and the bimolecular reactions with the water monomer, acetaldehyde and acetone under atmospheric conditions, *Physical Chemistry Chemical Physics*, 2015, **17**, 19862–19873.

[4] M. J. Newland, A. R. Rickard, M. S. Alam, L. Vereecken, A. Muñoz, M. Ródenas and W. J. Bloss, Kinetics of stabilised Criegee intermediates derived from alkene ozonolysis: reactions with SO_2 , H_2 O and decomposition under boundary layer conditions, *Physical Chemistry Chemical Physics*, 2015, **17**, 4076–4088.

[5] C. Robinson, L. Onel, J. Newman, R. Lade, K. Au, L. Sheps, D. E. Heard, P. W. Seakins, M. A. Blitz and D. Stone, Unimolecular Kinetics of Stabilized CH_3 CHOO Criegee Intermediates: *syn* -CH_3 CHOO Decomposition and *anti* -CH_3 CHOO Isomerization, *The Journal of Physical Chemistry A*, 2022, **126**, 6984–6994.

[6] Y. Fang, F. Liu, V. P. Barber, S. J. Klippenstein, A. B. McCoy and M. I. Lester, Communication: Real time observation of unimolecular decay of Criegee intermediates to OH radical products, *The Journal of Chemical Physics*, 2016, **144**, 061102.

[7] J. Peltola, P. Heinonen and A. Eskola, Direct Kinetic Measurements of a Cyclic Criegee Intermediate; Unimolecular Decomposition of *c*- $(CH_2\ )_5$ COO, *The Journal of Physical Chemistry Letters*, 2024, **15**, 5331–5336.

[8] S. Yu, S. Tong, M. Chen, H. Zhang, Y. Xu, Y. Guo and M. Ge, Characterization of Key Intermediates and Products from the Ozonolysis of Styrene-Like Compounds, *Environmental Science & Technology*, 2025, **59**, 11666–11676.

[9] B. Long, J. L. Bao and D. G. Truhlar, Rapid unimolecular reaction of stabilized Criegee intermediates and implications for atmospheric chemistry, *Nature Communications*, 2019, **10**, 2003.

[10] L. Wang and L. Wang, Mechanism of gas-phase ozonolysis of sabinene in the atmosphere, *Physical Chemistry Chemical Physics*, 2017, **19**, 24209–24218.
